# Spontaneous evisceration of infantile umbilical hernia

**DOI:** 10.1016/j.ijscr.2023.108352

**Published:** 2023-05-24

**Authors:** Ahmed Kamel Ali, Moataz Elagan, Fatma A. Monib, Tarek Abdelazeem Sabra

**Affiliations:** Faculty of Medicine, Assiut University, Egypt

**Keywords:** Herniorrhaphy, Pediatric patients, Spontaneous rupture, Umbilical hernia, Complicated paediatric umbilical hernia, Bowel evisceration with gangrene

## Abstract

**Introduction and importance:**

Infantile umbilical hernia is common in children. It has a regressive course in most cases. Conservative management is the standard in most cases before the age of 3 years unless there are complications such as incarceration, rupture with evisceration which are extremely rare and warrants emergency surgery.

**Case presentation:**

Our case was a full term 6-month-old male of normal birth weight with history of umbilical hernia but with no obvious risk factors to develop complications. The loops evisceration was spontaneous with a small umbilical skin damage. The poor parental consultation on early surgical management and delayed presentation of the infant after evisceration could be the possible risks for ischemic changes and shock state at the time of presentation, however, prompt medical resuscitation and surgical management relatively improved postoperative outcomes.

**Clinical discussion:**

Infantile umbilical hernia is considered one of the most encountered abnormalities of infancy. Most umbilical hernias are asymptomatic and discovered after birth. Complications of infantile umbilical hernia as incarceration or spontaneous evisceration are very rare but fatal. Certain factors increase the risk for developing spontaneous rupture of infantile umbilical hernia including the age of the infant or child, the defect size, umbilical sepsis or ulceration and any condition which raises intra-abdominal pressure, i.e., crying, coughing or positive ventilation.

**Conclusion:**

Although infantile umbilical hernia is clinically benign condition with a regressive course in majority of cases, the risk of rupture of an umbilical hernia is exceedingly rare in pediatric population; physicians should be warranted with the possible risk factors for spontaneous rupture and in these patients expedite surgical repair.

## Highlights

• Infantile umbilical hernia is one of the most common problems seen by pediatric surgeons.
• Spontaneous rupture and evisceration of bowel is extremely rare complication, but certain conditions are considered the main predisposing factors for this fatal complication including omphalitis, respiratory distress, distension of abdomen, ascites, umbilical ulceration and requirement of positive ventilation.
• Certain factors increase the risk for developing spontaneous rupture of infantile umbilical hernia including the age of the infant or child, the defect size, umbilical sepsis or ulceration and any condition which raises intra-abdominal pressure.
• The poor parental consultation on early surgical management and delayed presentation of the infant after evisceration could be the possible risks for ischemic changes and shock state at the time of presentation.

## 1 Introduction and importance

Infantile umbilical hernia is one of the most common problems seen by pediatric surgeons [1]. Race and prematurity can predispose to umbilical hernias. African infants are more likely to incur the defect than white infants and also, more than 80 % of infants weighing less than 1200 g have evidence of an umbilical hernia compared with 21 % of infants weighing over 2500 g at birth [2].

It has a regressive course in majority of cases and do not require operative closure [3]. Complications such as incarceration, rupture and evisceration are extremely rare and warrant emergency surgery [4].

Spontaneous rupture and evisceration of bowel is extremely rare complication, but certain conditions are considered the main predisposing factors for this fatal complication including omphalitis, respiratory distress, distension of abdomen, ascites, umbilical ulceration and requirement of positive ventilation [1,4,5].

The work has been reported in line with the SCARE criteria [6].

## 2 Case presentation

**Fig. 1:**
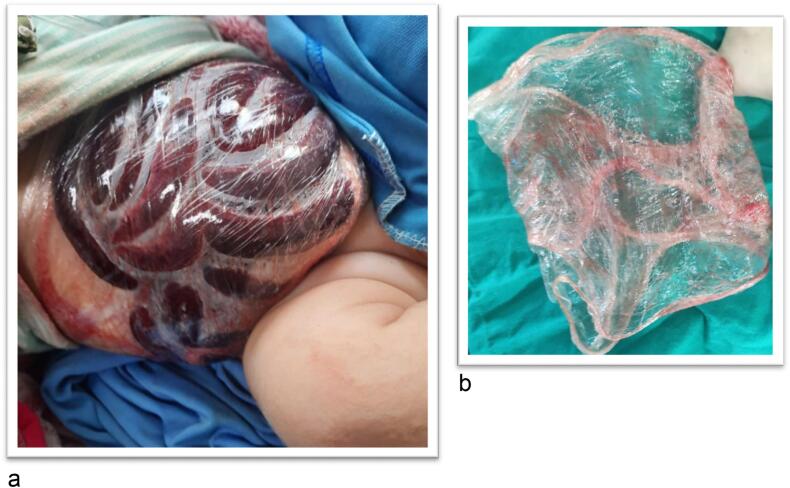
a: Eviscerated bowel loops with elastic sheet. b: The elastic coverage used to cover the eviscerated bowel loops.

**Fig. 2:**
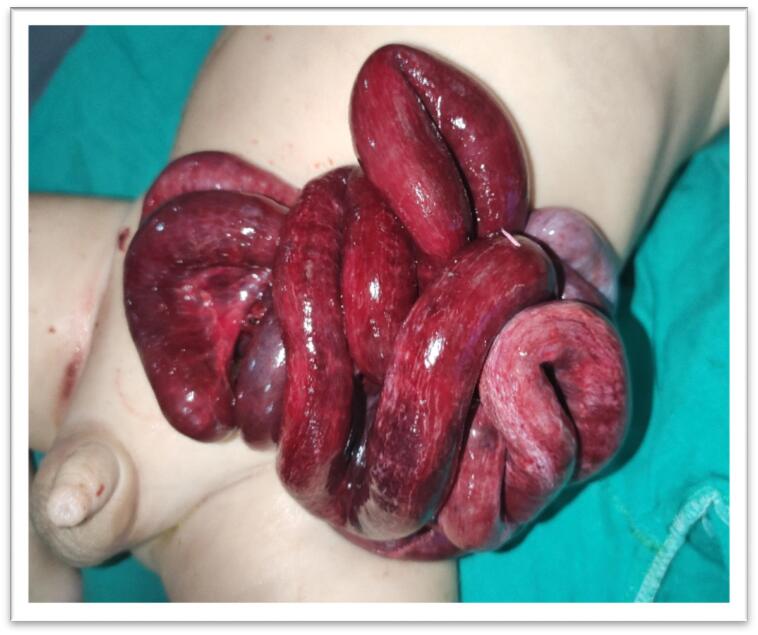
Clinically ischemic eviscerated bowel loops through small umbilical defect.

**Fig. 3:**
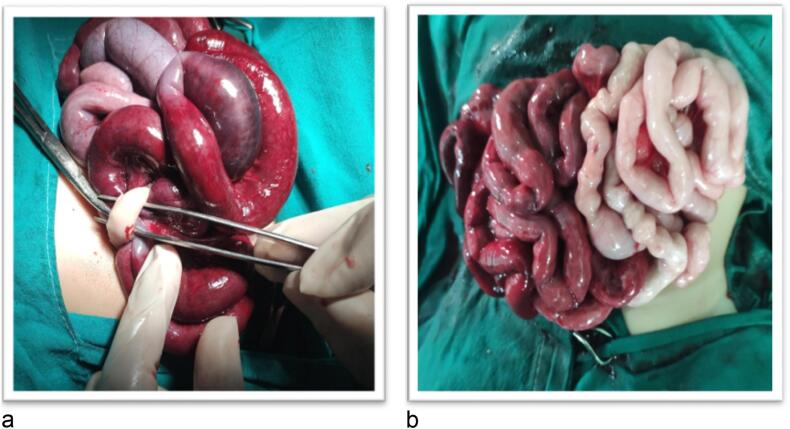
a: Eviscerated bowel loops with the tool in the skin defect at the summit of umbilicus. b: The discrepancy in color between viable and non-viable small intestine.

**Fig. 4:**
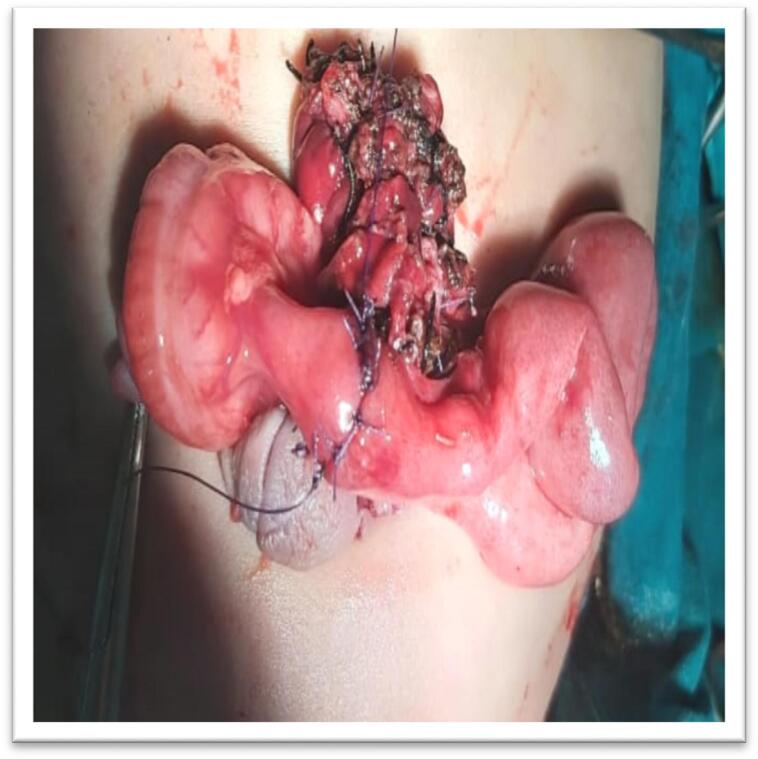
two layered jejunoileal anastomosis 5 cm far from ileocecal junction.

**Fig. 5:**
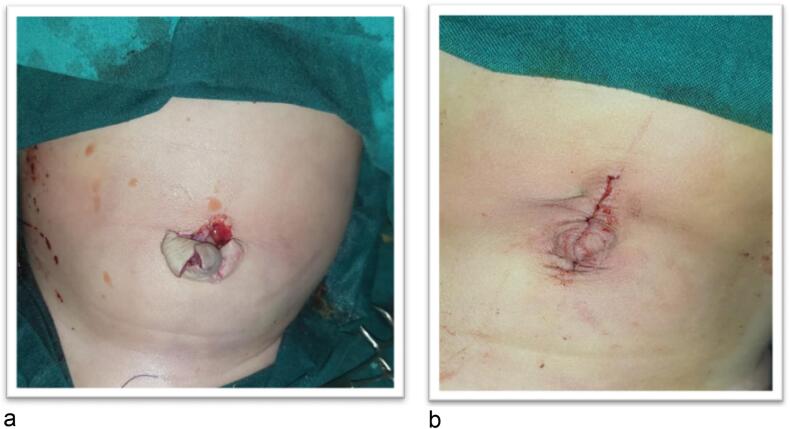
a: Facial defect after reduction of bowel loops. b: Skin closure with umbilicoplasty.

Male child aged 6 months old presented to the pediatric emergency room with eviscerated bowel loops on his abdomen covered with elastic sheet (Fig. 1a, b). First aid was administered at the neighborhood primary healthcare facility without attempting to reduce the hernia. Between the first sign of evisceration and the patient's admission to our hospital, more than 6 h elapsed. The patient has no significant medical history. On general examination the patient was shocked, pale, and dehydrated. His blood pressure was 70/40, heart rate was 170 beats per minute, respiratory rate was 36 breaths per minute, and his temperature was 36.5 °C. Abdominal examination revealed apparently ischemic small bowel loops (Fig. 2). A white blood cell count showed a leukocytosis of 21,500 cells/mm^3^ with a neutrophilic predominance of 18,500 cells/mm^3^. His hemoglobin level was 10 mg/dl. After proper resuscitation with intravenous fluids and parenteral antibiotics he was prepared for emergency surgery. Under general anesthesia examination revealed eviscerated ischemic small bowel loops through small facial defect at Linea alba beneath the umbilicus and skin defect at summit of umbilicus. (Fig. 3a, b). Via trans umbilical approach, exploration of bowel loops revealed perforated gangrenous small intestinal segment of about 100 cm length. Resection of gangrenous segment with primary two-layered re-anastomosis with Vicryl suture 3/0 was done (Fig. 4). Peritoneal lavage was done with insertion of pelvic drain. Herniorrhaphy was done with reconstruction of the umbilicus (Fig. 5a, b). The patient was transferred to intensive care unit postoperatively. On the 3rd day we introduced oral feeding. The patient was discharged on the 20th postoperative day in good clinical conditions.

The patient was regularly followed at outpatient clinic for next three months and he had no significant post operative complications.

## 3 Clinical discussion

Infantile umbilical hernia is considered one of the most encountered abnormalities of infancy. The direct cause of infantile umbilical hernia is thought to be failure of umbilical defect to close which is resulted in a defect in Linea alba covered by peritoneum posteriorly and intact skin anteriorly [3].

The incidence of an umbilical hernia is 10 to 30 % of all neonates and it is associated with race, birth weight, and certain syndromes as hypothyroidism, Down's syndrome, mucopolysaccharidosis, Beckwith-Wiedemann syndrome and exomphalos-macroglossia syndrome [1].

Most umbilical hernias are asymptomatic and discovered after birth. Most umbilical hernias spontaneously regress over time with tendency of the fascia to spontaneously close the defect [7].

Traditionally, there is a low risk of incarceration or strangulation, but the bowel or omentum may transiently appear in the hernia sac, causing pain, vomiting or constipation. Previous studies highlighted the variable rates of complications to alert physicians to the risk factors that may trigger earlier surgical response or encourage proper consultation of parents to take a surgical decision before complications [8].

There are major indications for surgical closure including hernias with a diameter more than 2 cm at the age of 2–3 years, persistent hernia at the age of 4–5 years and in those with symptoms of incarceration or recurring pain [1].

Complications of infantile umbilical hernia as incarceration or spontaneous evisceration are very rare but fatal [5]. Certain factors increase the risk for developing spontaneous rupture of infantile umbilical hernia including the age of the infant or child, the defect size, umbilical sepsis or ulceration and any condition which raises intra-abdominal pressure, i.e., crying, coughing or positive ventilation [1]. A case report published in 2006 included the data of eight other cases dating back to the fifties and discussed similar risk factors [9].

A recent report of a 40-day-old female infant with a history of umbilical hernia, was a full term and of normal birth weight presented to the ER with bowel loops exposed on the abdominal wall through the umbilicus for 15 h with some ischemic loops. Five days earlier, the infant had diarrhea that was managed by natural remedies and Asafoetida paste on the umbilicus which is suspected to cause irritation and local ulceration that led to this evisceration episode as there was no history of skin damage before applying that paste. This further emphasizes the damage of skin over the umbilical hernia as a sole risk factor for evisceration [10].

Our case was a full term 6-month-old male of normal birth weight with history of umbilical hernia but with no obvious risk factors to develop complications. The loops evisceration was spontaneous with a small umbilical skin damage. The poor parental consultation on early surgical management and delayed presentation of the infant after evisceration could be the possible risks for ischemic changes and shock state at the time of presentation, however, prompt medical resuscitation and surgical management relatively improved postoperative outcomes.

## 4 Conclusion

Although infantile umbilical hernia is clinically benign condition with a regressive course in majority of cases, the risk of rupture of an umbilical hernia is exceedingly rare in pediatric population; we the physician should be warranted with the potential risk factors for spontaneous rupture and in these patients expedite surgical repair.

## Ethical approval

Our institution does not require ethical approval for reporting individual cases or case series.

## Funding

The author(s) received no financial support for the research, authorship and/or publication of this article.

## Consent

Written informed consent was obtained from the patient's parents/legal guardian for publication and any accompanying images. A copy of the written consent is available for review by the Editor-in-Chief of this journal on request.

## Provenance and peer review

Not commissioned, externally peer reviewed.

## CRediT authorship contribution statement

Ahmed Kamel Ali contributed to management and wrote the manuscript.

Tarek Abdelazeem Sabra and Moataz Elagan contributed to acquisition of data and revision of manuscript.

Ahmed Kamel Ali and Tarek Abdelazeem Sabra were the surgeon who operated this case. Moataz Elagan was the anesthesiologist. Ahmed Kamel Ali and Fatma Monib critically revised the manuscript. All authors read and approved the final manuscript.

## Guarantor

Ahmed Kamel Ali.

## Registration of research studies

1. Name of the registry: Clinicaltrials.gov –for all human studies
2. Unique identifying number or registration ID NCT05852535
3. Hyperlink to your specific registration (must be publicly accessible and will be checked): https://clinicaltrials.gov/ct2/show/NCT05852535

## Declaration of competing interest

The author(s) declared no potential conflicts of interest with respect to the research, authorship and/or publication of this article.

## Data Availability

The data that support the findings of this study are available from the corresponding author upon reasonable request.
